# Metabolic remodeling of pyrimidine synthesis pathway and serine synthesis pathway in human glioblastoma

**DOI:** 10.1038/s41598-022-20613-w

**Published:** 2022-09-29

**Authors:** Akira Nakamizo, Yuichiro Miyamatsu, Haruka Hirose, Toshiyuki Amano, Satoshi Matsuo, Minako Fujiwara, Teppei Shimamura, Koji Yoshimoto

**Affiliations:** 1 Department of Neurosurgery, Clinical Research Institute, National Hospital Organization, Kyushu Medical Center, Fukuoka, Japan; 2 Division of Systems Biology, Graduate School of Medicine, Nagoya University, Nagoya, Japan; 3 Department of Pathology, Clinical Research Institute, National Hospital Organization, Kyushu Medical Center, Fukuoka, Japan; 4 Department of Neurosurgery, Graduate School of Medical Sciences, Kyushu University, 3-1-1 Maidashi, Higashi-Ku, Fukuoka, 812-8582 Japan

**Keywords:** CNS cancer, Cancer metabolism

## Abstract

Glioblastoma is the most common brain tumor with dismal outcomes in adults. Metabolic remodeling is now widely acknowledged as a hallmark of cancer cells, but glioblastoma-specific metabolic pathways remain unclear. Here we show, using a large-scale targeted proteomics platform and integrated molecular pathway-level analysis tool, that the de novo pyrimidine synthesis pathway and serine synthesis pathway (SSP) are the major enriched pathways in vivo for patients with glioblastoma. Among the enzymes associated with nucleotide synthesis, RRM1 and NME1 are significantly upregulated in glioblastoma. In the SSP, SHMT2 and PSPH are upregulated but the upstream enzyme PSAT1 is downregulated in glioblastoma. Kaplan–Meier curves of overall survival for the GSE16011 and The Cancer Genome Atlas datasets revealed that high SSP activity correlated with poor outcome. Enzymes relating to the pyrimidine synthesis pathway and SSP might offer therapeutic targets for new glioblastoma treatments.

## Introduction

Glioblastoma is the most common WHO grade IV primary brain tumor in adults^1^, and shows a dismal overall survival of 14.6–21 months^2,3^. Over 90% of patients are dead within 3 years of diagnosis^4^. Developing new treatment strategies for this pathology represents an urgent issue, with glioblastoma treatments currently limited to surgical resection, radiotherapy, and chemotherapy (typically temozolomide) for many years^5^. Current treatments for other cancers include cytotoxic chemotherapy^6^, cell signaling inhibition^6–8^, immunotherapy^9^, and metabolic approaches^10^. Among these, metabolic approaches are founded upon presumed dissimilarities in the metabolism of cancer cells and normal cells^10^ which would provide potential targets for therapeutic inhibition. The glioblastoma-specific metabolism has not yet been sufficiently studied to allow such approaches.

A major task of metabolism in normal cells is to maintain homeostasis using adenosine triphosphate (ATP) as energy for fueling housekeeping processes via oxidative phosphorylation. In contrast, the metabolism in cancer cells is shifted toward producing biomass for building new cells and surviving the tumor microenvironment of limited oxygen and nutrients, nucleotide synthesis for genome replication, amino acid synthesis for proteins, lipid synthesis for membranes, and glutathione synthesis for redox balance^11–13^. Metabolic remodeling is now widely acknowledged as a hallmark of cancer cells^14^. A more detailed understanding of global metabolic networks holds promise for the treatment of cancer patients^13^. However, precise and comprehensive measurement of many metabolizing enzymes and identification of the metabolic pathways representing the key differences between cancer cells and normal cells by searching for individual enzymes have proven difficult.

Recently, a large-scale targeted proteomics platform, iMPAQT (in vitro proteome-assisted multiple reaction monitoring (MRM) for protein absolute quantification) has been introduced to provide simultaneous global measurements of the abundance of proteins^15^. Moreover, the web interface IMPaLA (integrated molecular pathway-level analysis) for joint pathway analysis of transcriptomics, proteomics, and metabolomics data has been developed^16^ to interpret such post-genomic data at a higher level than that of individual biomolecules^17,18^. The current version 13 was released in June 2021 and performs over-representation or enrichment analysis using 4813 pre-annotated pathways from 12 databases (available at http://impala.molgen.mpg.de).

Here we investigated differences in the metabolic pathway networks between glioblastoma and near-normal brain in vivo in patients using iMPAQT and IMPaLA.

## Results

### Glioblastoma tissue samples

**Figure 1:**
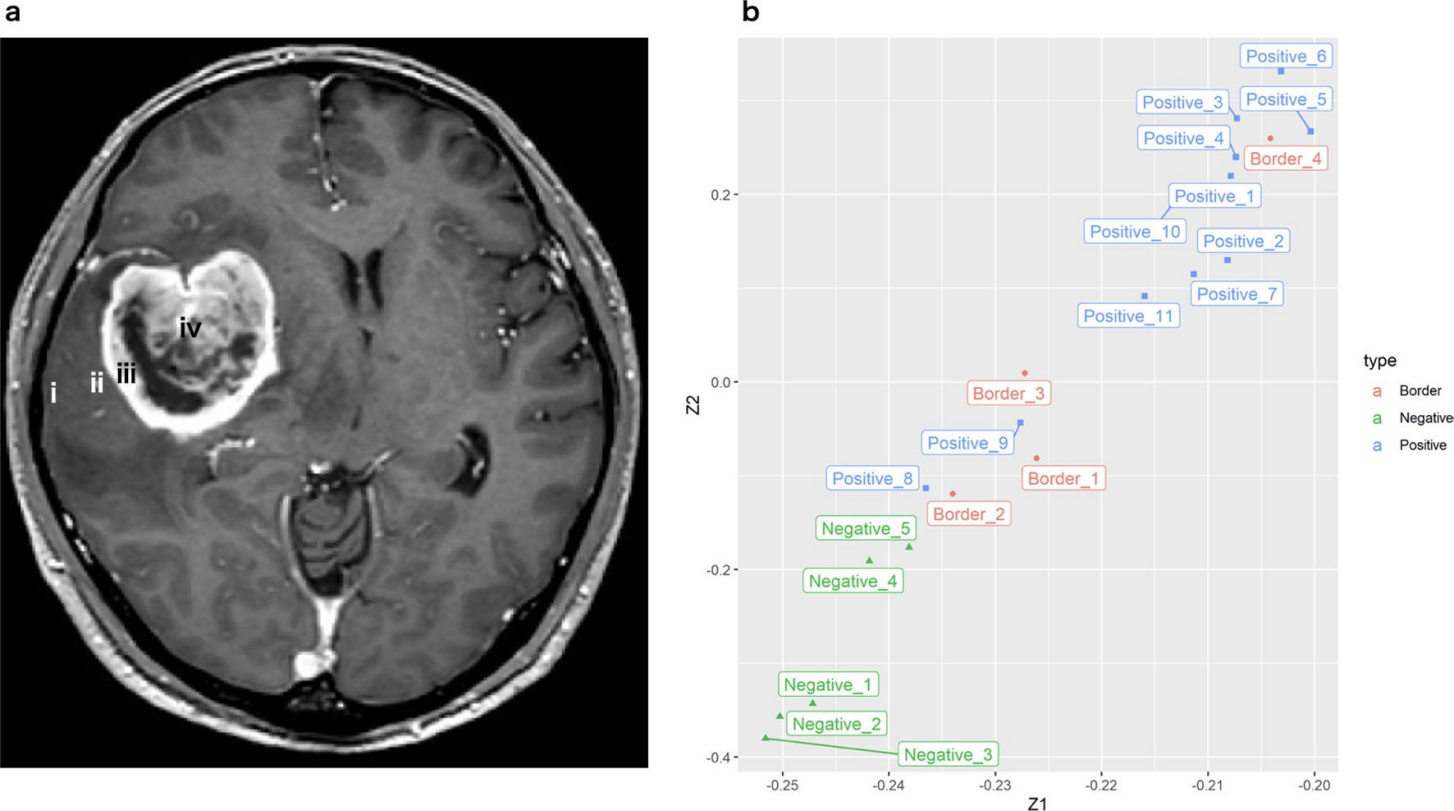
(**a**) Magnetic resonance imaging shows tissue sample collection sites: (i) far from the tumor; (ii) just outside the tumor; (iii) at the tumor periphery; and (iv) at the tumor core. (**b**) The scatterplot of the first and second principal component loadings for GBM and near-normal brain tissues. The x- and y-axis (Z1 and Z2) represent the loadings of the first and second principal components (PC1 and PC2), and colors indicate Positive (blue), Border (red), or Negative (green), respectively. The sum of variance ratios for PC1 and PC2 are 0.90 and 0.94, respectively.

Glioblastoma tissue samples were obtained from five adult patients with the assistance of an intraoperative 5-aminolevulinic acid (5-ALA)-induced fluorescence and neuronavigation system. Four samples per patient were collected from the brain where possible, as follows: (i) far from the tumor with 5-ALA non-fluorescence; (ii) just outside the tumor with slight 5-ALA fluorescence; (iii) at the tumor periphery with 5-ALA fluorescence; and (iv) at the tumor center with 5-ALA fluorescence (Fig. 1a). Finally, collected samples were re-organized according to the findings from 5-ALA fluorescence and histopathological examinations, as follows: Negative (n = 5); Borderline (n = 4), and Positive (n = 11) (Table 1). A normal brain structure was maintained in ≥ 90%, 70–90%, or 0% in Negative, Border, and Positive regions, respectively. Principal component analysis score plots showed clear cluster separation between Positive (blue) and Negative (green) regions (Fig. 1b). Positive_8 and Positive_9 obtained from Patient 4 were located closer to Border_1, _2, and _3 than other Positives. This inter-patient difference was consistent with the low Ki67 labeling index in Patient 4. Positives obtained from the same patient were located close to each other and their Ki67 labeling indices were almost the same (Positive_1 and _2, Positive_3 to _5, Positive _8 and _9, and Positive_10 and _11). This suggested that the intratumoral difference was relatively small.

**Table 1:** Patient characteristics.

Patient	Age/sex	Tumor location	Samples	Ki67 labeling index (%)	Collection sites in Fig. 1a
1	79/male	Temporal lobe	Positive_1	50	(iii)
			Positive_2	70	(iv)
			Border_1	30	(ii)
			Negative_1	5	(i)
2	61/male	Insular cortex	Positive_3	17	(ii)
			Positive_4	27	(iii)
			Positive_5	40	(iv)
			Negative_2	7	(i)
3	65/male	Temporal lobe	Positive_6	48	(iii)
			Positive_7	60	(iv)
			Border_2	7	(ii)
			Negative_3	5	(i)
4	67/male	Temporal lobe	Positive_8	13	(iii)
			Positive_9	17	(iv)
			Border_3	10	(ii)
			Negative_4	8	(i)
5	82/male	Frontal lobe	Positive_10	53	(iii)
			Positive_11	55	(iv)
			Border_4	40	(ii)
			Negative_5	7	(i)

### Global view of metabolism in glioblastoma

**Figure 2:**
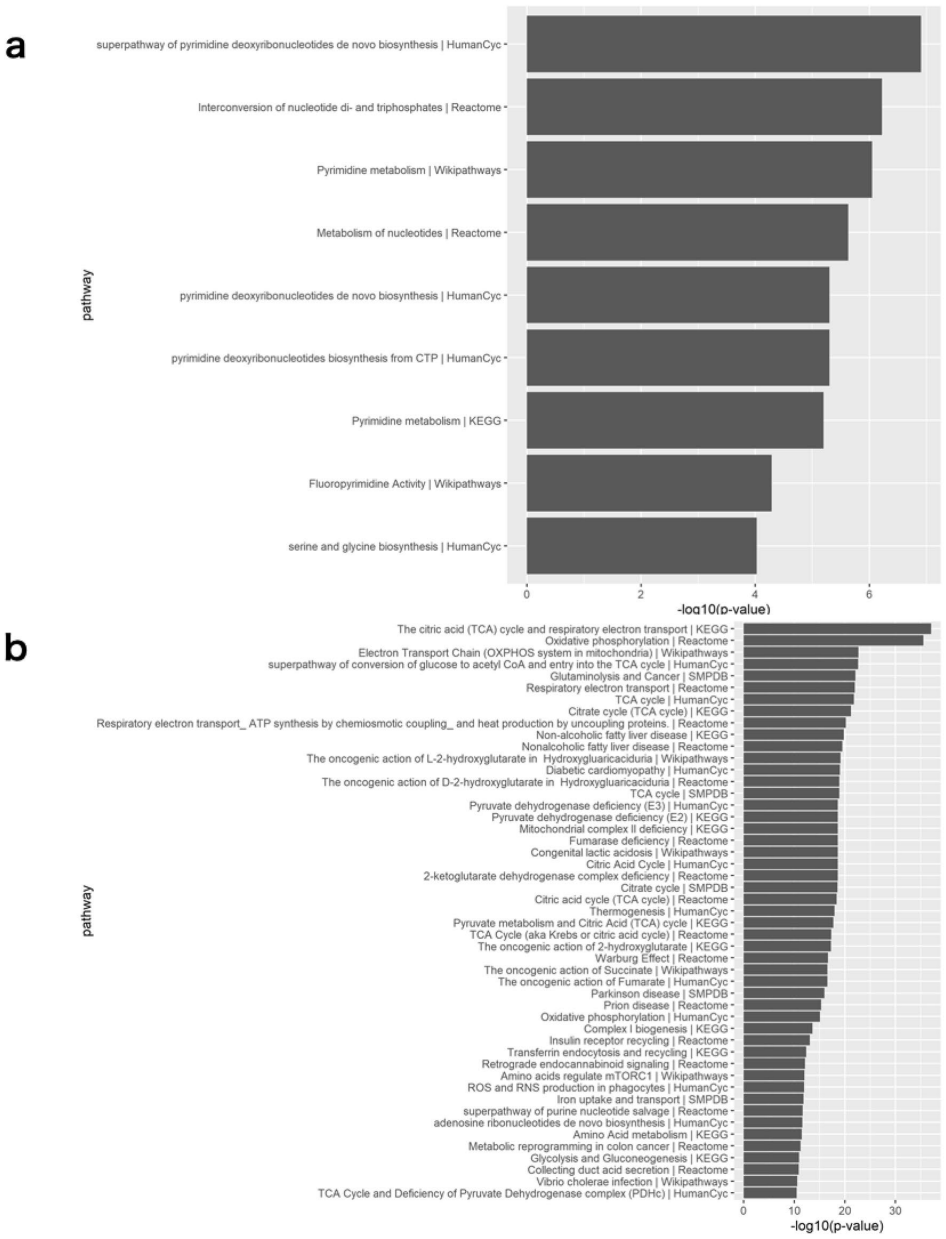
Enriched molecular pathways with pathway overrepresentation analysis in 32 enzymes with 1.5-fold higher expressed and p-value < 0.05 and 67 enzymes with 0.67 (= 1/1.5)-fold lower expressed and p-value < 0.05 in the Positive compared to the Negative through the use of IMPaLA. The protein data from a gene by sample matrix is transformed to a gene-set by sample matrix with gene set variation analysis (R package gsva version 1.38.0). (**a**) Upregulated pathway analysis. (**b**) Downregulated pathway analysis.

**Figure 3:**
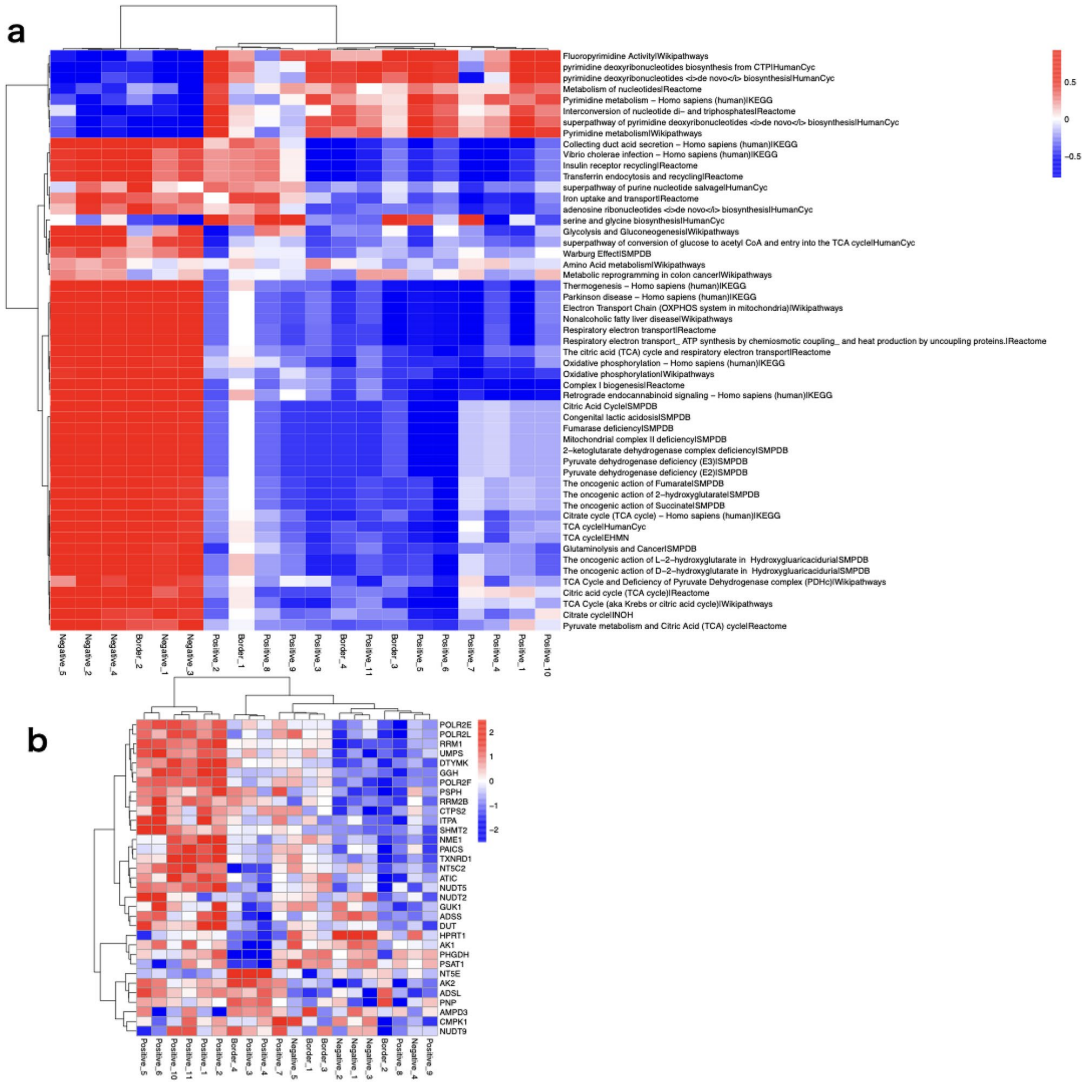
(**a**) Heatmap of the enrichment score matrix for 20 tissues and 53 metabolic pathways with fewer than 300 genes and p-values of less than 10^−4^ in pathway over-representation analysis (R package pheatmap version 1.0.12). The color intensity indicates the level of enrichment score of each pathway which was calculated by gene set variation analysis. (**b**) Heat map of the 33 enzymes in the enriched metabolic pathway. The color intensity indicates the expression level of each enzyme.

We applied iMPAQT to tissue samples to comprehensively measure the abundance of 352 metabolizing enzymes. Of these, we successfully quantified the abundance of 255 enzymes. The results of pathway overrepresentation analysis revealed that the pathways upregulated in Positive compared to Negative were significantly enriched in the following biological processes: ‘pyrimidine biosynthesis’, ‘nucleotide metabolism’, and ‘serine and glycine biosynthesis’ (Fig. 2a). On the other hand, downregulated pathway analysis demonstrated downregulation of ‘the citric acid (TCA) cycle’ and ‘oxidative phosphorylation’ in Positive compared to Negative (Fig. 2b). A heatmap of enriched pathways showed good differentiation between Positive and Negative (Fig. 3a). A heatmap of enzymes included in the enriched metabolic pathways revealed that many enzymes relating to nucleotide synthesis and SSP were upregulated in Positive (Fig. 3b).

### Nucleotide synthesis in glioblastoma

**Figure 4:**
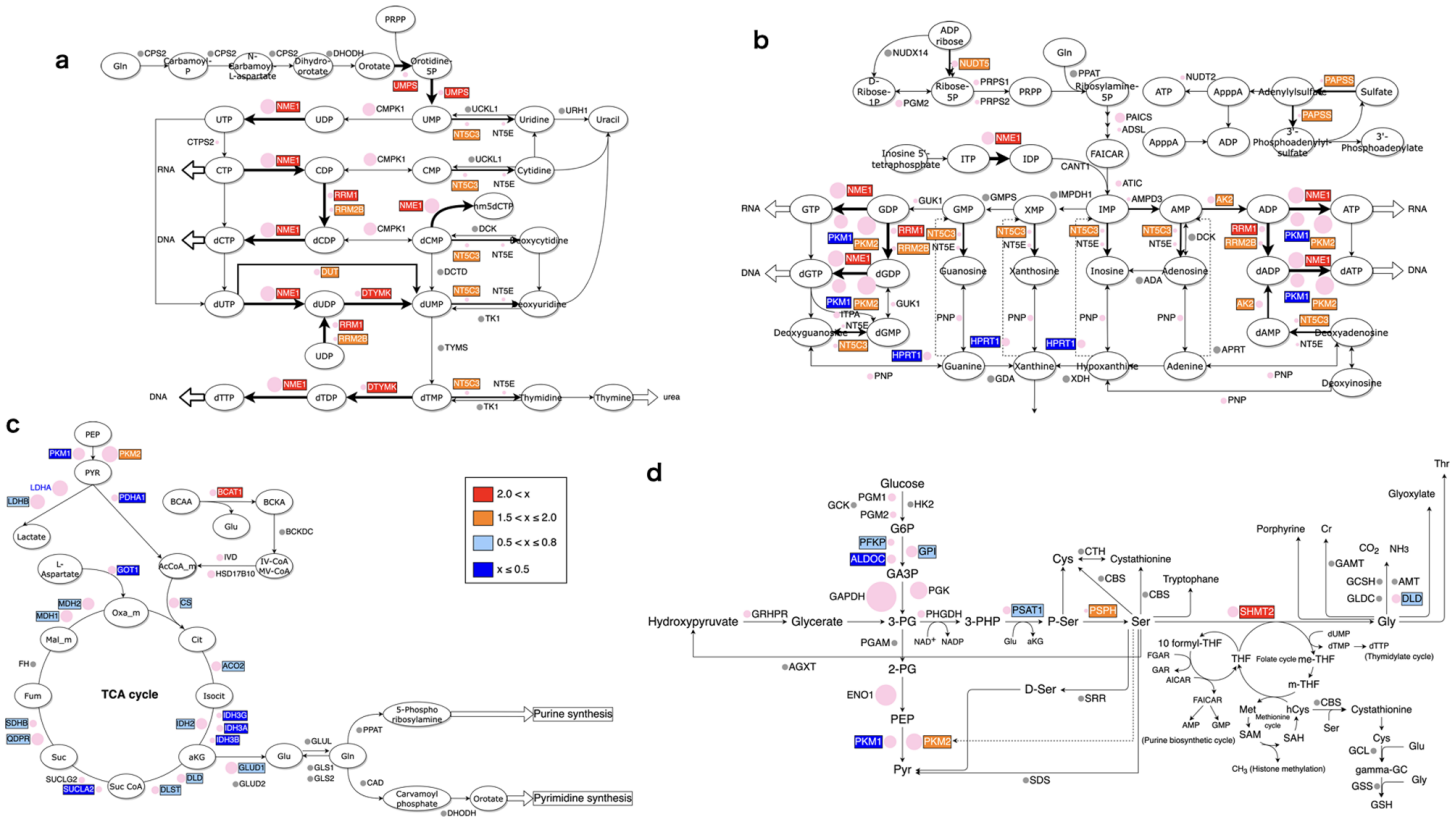
iMPAQT-determined changes in the abundance of enzymes. The size of each pink circle represents the abundance of the indicated enzyme. Grey circle means the abundance of the enzyme is either not detectable or not measured. The fold change is Positive vs Negative. (**a**) Pyrimidine synthesis pathway. (**b**) Purine synthesis pathway. (**c**) TCA cycle. (**d**) Serine synthesis pathway.

To better characterize upregulated enzymes in nucleotide synthesis pathways and the SSP, we plotted those pathways with abundance and fold changes of enzymes in Positive compared to Negative. Many enzymes in the de novo pyrimidine synthesis pathway (Fig. 4a) and purine synthesis pathway (Fig. 4b) were upregulated in Positive; NME1, RRM1, RRM2B, and NT5C3 were significantly upregulated in both pyrimidine and purine synthesis pathways. PKM2 was upregulated in the purine synthesis pathway, while hypoxanthine phosphoribosyl transferase 1 (HPRT1), which plays a central role in purine nucleotide generation through the purine salvage pathway, was downregulated in Positive. In contrast, many enzymes relating to the TCA cycle other than PKM2 were downregulated in Positive compared to Negative (Fig. 4c).

### SSP in glioblastoma

In the SSP, several rate-limiting enzymes were affected in Positive compared to Negative (Fig. 4d). Serine hydroxymethyltransferase (SHMT2), which converts serine to glycine, and phosphoserine phosphatase (PSPH), which converts phosphoserine to serine, were upregulated in Positive. The upstream rate-limiting enzyme phosphoserine aminotransferase (PSAT)1 was downregulated and 3-phosphoglycerate (3-PG) dehydrogenase (PHGDH), which diverts carbon flux from glycolysis to the SSP, was unaffected in Positive.

### Survival and SSP in glioblastoma

**Figure 5:**
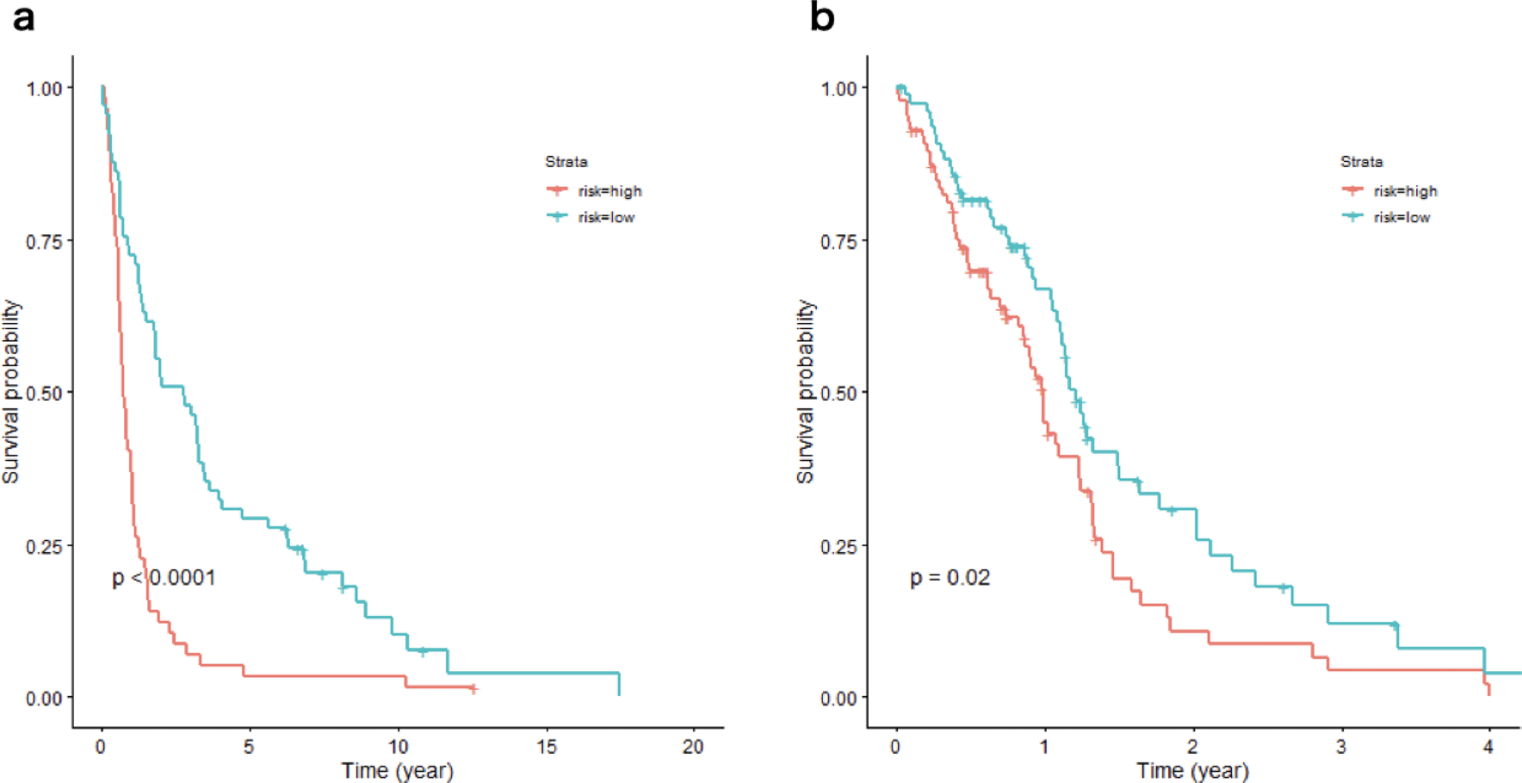
Overall Kaplan–Meier curves of high risk and low risk groups in (**a**) the GSE16011 glioma patients and (**b**) the TCGA glioblastoma patients with Cox proportional hazard modeling using 7 gene expressions (*SHMT1*, *SHMT2*, *VPS29*, *PSAT1*, *SRR*, *PHGDH*, and *PSPH*) in serine and glycine biosynthesis pathway. Overall patient survival was determined in patients with high risk (log risk > 0) and low risk (log risk < 0) categorized by the Cox proportional hazard model based on expression of the 7 genes. The p-value was calculated by the log-rank test to evaluate whether two survival curves were statistically significantly different.

Kaplan–Meier curves for the overall survival of GSE16011 glioma patients (Fig. 5a) and The Cancer Genome Atlas (TCGA) glioblastoma patients (Fig. 5b) revealed that high SSP activity correlated with poor outcomes and had high prognostic significance.

Unfortunately, the abundances of PPAT, IMPDH1, DCK, UCKL1, TK1, CBS, CTH, and GAMT could not be detected by iMPAQT assay, and several other enzymes were not measured in this analysis.

## Discussion

Our results suggest that the metabolism of glioblastoma was highly dependent on the pyrimidine synthesis pathway and SSP. Pyrimidine metabolism is disrupted during the development of many cancers^19^ and pyrimidine metabolic rate-limiting enzymes are markedly expressed in bladder cancer, breast cancer, colon cancer, liver cancer, and lung cancer^20,21^. However, the preferred precursors have not yet been defined in human cancer tissues^22^. We showed that RRM1 and NME1 were significantly upregulated in both pyrimidine and purine synthesis pathways in glioblastoma, elevated more than twofold compared to near-normal brain, suggesting that these enzymes might offer therapeutic targets for the treatment of glioblastoma. RRM1 is the large regulatory subunit of ribonucleotide reductase and combines with the small catalytic subunit RRM2; these two subunits form the active enzyme that is responsible for de novo deoxyribonucleotides synthesis^23^. Higher RRM1 expression was associated with shorter survival in non-small cell lung cancer patients treated with gemcitabine-based therapies^24^, but with better outcomes in malignant pleural mesothelioma patients treated using different chemotherapy regimen^25^. RRM1 might play different tissue-specific functions depending on cancer types. On the other hand, NME1, the first identified metastasis suppressor gene, inhibited metastasis when overexpressed in mono-layered melanoma cell lines^26^, but promoted the proliferation of melanoma sphere cells and lung-colonizing activities, indicating that the description of NME1 merely as a metastasis suppressor appears to need refinement^27^. This is consistent with the fact that the product of the *NME1* gene is a nucleoside diphosphate kinase involved in cell proliferation^28,29^. Upregulated NME1 expression associated with poor outcome in neuroblastoma^30,31^ and some types of leukemia and lymphoma^32^. PKM2 was also upregulated in the purine synthesis pathway of glioblastoma in our study. PKM2 is associated with proliferating cells^33,34^. PKM2 phosphorylates GDP to produce GTP for the guanine nucleotide and catalyzes the transphosphorylation between phosphoenolpyruvate (PEP) and ADP to produce pyruvate and ATP^35^.

The SSP has been noticed as a potential therapeutic target because increased activity of the SSP is associated with worse outcomes in breast cancer, melanoma, and non-small cell lung cancer^36,37^. In glioma cell lines, proliferation was impaired by the inhibition of this pathway under conditions of nutrient and oxygen deprivation^38^. Serine is a central node for the biosynthesis of many molecules, including glycine, cysteine, sphingolipids, and phospholipids^13^. Serine can be obtained either from extracellular serine through amino acid transporters^39^ or by intracellular de novo biosynthesis from the glycolytic intermediate 3-PG^40^. Despite abundant extracellular serine, some cancer cells actively synthesize serine via the SSP, suggesting that de novo serine synthesis contributes to cancer cells more than provision of serine itself^41,42^. One important role of SSP is the contribution to nucleotide synthesis. The biosynthesis of either pyrimidine or purine nucleotides requires cofactors generated through one-carbon metabolism, which is closely linked to the SSP^43^. The conversion of serine to glycine donates a one-carbon unit to tetrahydrofolate (THF) to produce 5,10-methylene-THF, a precursor of other folate species that play important roles in pyrimidine and purine biosynthesis^13,43–45^. This 5,10-methylene-THF acts as a one-carbon unit for the first step of pyrimidine biosynthesis catalyzed by thymidylate synthase^43^. In purine biosynthesis, one-carbon units from the folate pool convert the precursor GAR to AMP or GMP^13^, and 10-formyl-THF converts AICAR to FAICAR^46^. The folate cycle also regenerates S-adenosylmethionine, which is a sole methyl donor for both DNA and histone methylation reactions^13^, providing a close connection between cellular metabolism and epigenetic control of gene expression^5,13^. In addition, the one-carbon pool contributes to the maintenance of redox homeostasis by supplying NADPH, which increases cellular antioxidative ability by revitalizing the cellular pool of reduced glutathione and thioredoxin^47^. The SSP thus provides a survival advantage for cancers, particularly in ischemic environments.

Our results showed that SHMT2 and PSPH were upregulated in SSP, while their upstream enzymes PSAT1 or PHGDH were downregulated or unaffected in glioblastoma, suggesting that the SSP in glioblastoma might be independent of carbon flux from glycolysis. The first rate-limiting step for the SSP to divert carbon from glycolysis to this pathway is catalyzed by PHGDH^48^, followed by PSAT1 and PSPH^13^. Whether PHGDH is required for cancer proliferation remains controversial, as some studies have shown that PHGDH knockdown suppressed tumor growth in breast cancer cells and non-small cell lung cancer cells^36,37^, while another study revealed that PHGDH-knockdown had no effect on tumor maintenance in breast cancer cells^49^. In gliomas, one study showed that PHGDH expression was associated with overall survival^50^, while another demonstrated that PHGDH expression was present in not all glioblastoma cell lines^38^. PHGDH inhibition may thus suppress proliferation of glioblastoma, but identifying patients likely to respond to PHGDH inhibition may be important because our results showed that PHGDH was unaffected in glioblastoma compared to near-normal brain. PSPH catalyzes the hydrolysis of 3-PS to produce serine. PSPH exhibits different expression patterns in many cancers and has been associated with the poor prognosis of patients with various cancers, including gastric cancer^51^. However, little is known about the role of PSPH in patients with glioblastoma.

SHMT2 is the mitochondrial isoform that catalyzes the conversion of serine to glycine and is essential for providing glycine in proliferating cancer cells^52^. SHMT2 was expressed in the pseudopalisading cells of human glioblastoma samples, and proliferation of the LN229 glioblastoma cell line was impaired or enhanced by SHMT2 suppression or overexpression under hypoxic conditions^46^. Serine consumption by elevated SHMT2 can limit PKM2 activity^46,53^ because PKM2 is activated by intracellular serine, fructose bisphosphate, and SAICAR^54–56^. Reduced PKM2 activity can increase the levels of PEP and citrate, resulting in more glucose carbon flux into the SSP^13,57,58^. In our study, both SHMT2 and PKM2 were upregulated in glioblastoma compared to near-normal brain. Sufficient extracellular serine uptake might diminish intracellular serine consumption by SHMT2, or PKM2 might have been more activated before SHMT2 antagonized PKM2 activity, but the underlying mechanism remains unclear.

We identified differences in metabolic pathways between glioblastoma and near-normal brain in vivo in patients using a joint pathway enrichment analysis of proteomics data. This study showed some drawbacks. We did not measure all enzymes relating to nucleotide synthesis pathways and the SSP because the initial aim of this study was to provide a global view of glioblastoma-specific metabolism. The iMPAQT could not quantify the abundance of several enzymes, including PPAT and GLS1, from our tissue samples. These enzymes contribute to the malignant progression of cancer and have been successfully quantified by iMPAQT in cultured cell lines^59^. This discrepancy might have been caused by the fact that the amounts of several rate-limiting enzymes were markedly lower than those of enzymes mediating later steps of metabolic pathways^15^ and significantly lower in tissue samples than in cultured cells. RNA sequencing might be helpful to analyze the expression of enzymes not detected by iMPAQT or regulated predominantly at translational or post-translational levels^15^. This study had experimental limitations. The bulk cell analysis is ineffective in identifying the intratumoral and interpatient differences at the single cell level. Further studies are needed to clarify the intratumoral heterogeneity and interpatient variability in glioblastomas.

In summary, we quantified the abundance of 255 metabolic enzymes in tissue samples obtained from glioblastoma patients. Pyrimidine synthesis pathway and the SSP were enriched in glioblastoma. RRM1 and NME1 were significantly upregulated in both pyrimidine and purine synthesis pathways. SHMT2 and PSPH were upregulated and the upstream enzyme PSAT1 was downregulated, suggesting that SHMT2 and PSPH play key roles in activation of the SSP in glioblastoma. In conclusion, these enzymes might offer potential therapeutic targets for the establishment of new glioblastoma treatments.

## Method

### Glioblastoma tissue samples

This prospective study included five glioblastoma patients (5 men; age range, 61–82 years) who had never received any treatments for their brain tumors. All patients underwent tumor resection in the Department of Neurosurgery at National Hospital Organization Kyushu Medical Center. This study was approved by the National Hospital Organization Kyushu Medical Center Ethical Review Board and performed in accordance with the relevant guidelines and regulations, and all patients provided written informed consent prior to enrolment. Contrast-enhanced T1-weighted imaging was obtained for the navigation system. All patients received an oral solution of 5-ALA at 20 mg/kg body weight 3 h before induction of anesthesia for a standard protocol of glioblastoma resection. After formalin fixation and paraffin embedding, all patients were confirmed to have ‘Glioblastoma, IDH-wildtype’ according to the current WHO classification of central nervous system tumors.

### Sample preparation for iMPAQT

This study employed the iMPAQT assay to perform simultaneous global analysis for absolute quantification of protein expression. Analysis was performed as previously described^15^. In brief, frozen tissue was crushed with a bead shocker and lysed in 100 μL lysis buffer per 10 mg of tissue powder. The sample was diluted with an equal amount of water. Protein concentrations were determined with bicinchoninic acid assays (Thermo Fisher). To block cysteine/cysteine residues, portions including 200 μg of protein was treated with 5.0 mM Tris(2-carboxyethyl)phosphine hydrochloride for 30 min at 37 °C, then alkylated with 10 mM 2-iodoacetoamide for 30 min at room temperature. After acetone precipitation, the resulting pellet was resuspended in 100 μL 0.5 M triethylammonium bicarbonate digestion buffer. Each sample was digested with 2 μg of lysyl-endopeptidase for 3 h at 37 °C. After samples were further digested with 4 μg of trypsin for 14 h at 37 °C, the resulting digests were freeze-dried and labeled with the mTRAQ Δ0(light) reagent. Each sample was spiked with synthetic peptides (Funakoshi and GenScript) for internal standard which treated reductive alkylation and mTRAQ Δ4(heavy) labeling. Pretreatment and experiments using mass spectrometry were performed by an external service provider (Kyushu Pro Search LLP, Fukuoka, Japan).

### MRM analysis

MRM analysis was performed with a QTRAP6500 instrument (SCIEX) operated in positive-ion mode. Typical parameters were set as follows: spray voltage, 5500 V; curtain gas setting, 30; collision gas setting, 12; ion-source gas-1 setting, 40, ion-source gas-2 setting, 60; and interface-heater temperature, 350 °C. Collision energy (CE) was calculated using the following formulae: CE = (0.044 × *m*/*z*1) + 5.5 and CE = (0.051 × *m*/*z*1) + 0.5 (where *m*/*z*1 is the *m*/*z* of the precursor ion) for double- and triple-charged precursor ions, respectively. Collision-cell exit potential (CXP) was calculated according to the formula: CXP = (0.0391 × *m*/*z*2) − 2.2334, where *m*/*z*2 is the *m*/*z* of the fragment ion. The declustering potential (DP) was set to 50, and the entrance potential (EP) was set to 10. Resolution for Q1 and Q3 was set to ‘unit’ (half-maximal peak width, 0.7 m/z). The scheduled MRM option was used for all data acquisitions, with a target scan time of 2.0 s and MRM detection windows of 150–300 s for verification of MRM assays. After the determination of retention time, the detection window was narrowed to 160 s. Typically, 0.2 µl of internal standard was added to 1 µg of sample digest, and 10 µg of the resulting mixture was applied to the column. MRM analysis was also performed by Kyushu Pro Search LLP.

### Statistical methods

We used principal component analysis to project a protein expression dataset of 255 enzymes for glioblastoma and near-normal brain tissues onto the first two components. Lower-dimensional representations of protein expression measurements from these tissues were shown by a scatterplot of the first two principal component scores (Fig. 1b). Comparisons between two groups in Positive and Negative were analyzed using Welch's t test (see Supplementary Table S1 online).

### Metabolic pathway analysis

Thirty-two enzymes with 1.5-fold higher expressed and p-value < 0.05 and 67 enzymes with 0.67(= 1/1.5)-fold lower expressed and p-value < 0.05 in the Positive compared to the Negative were analyzed with pathway over-representation analysis through the use of IMPaLA. The detailed results of pathway enrichment analysis were summarized in Supplementary Table S2 online. We transformed the protein data from a gene by sample matrix to a gene-set by sample matrix with gene set variation analysis^60^, which was implemented in the R package gsva (version 1.38.0). This transformation allows the evaluation of pathway enrichment for each sample. The heatmap of the enrichment score matrix for 20 tissues and 53 metabolic pathways, as well as the heatmap of the 33 enzymes of the enriched metabolic pathways, were generated with the R package pheatmap (version 1.0.12).

### Survival analysis

For two gene expression datasets in glioblastoma cohort studies^61,62^ survival analysis using 7 genes (*SHMT1*, *SHMT2*, *VPS29*, *PSAT1*, *SRR*, *PHGDH*, and *PSPH*) in the serine and glycine biosynthesis pathways from the Encyclopedia of Human Genes and Metabolism (https://humancyc.org) was performed by Cox proportional hazard modeling using the R package survival (version 3.2.7). The coefficients of each gene in the model in two cohort studies were summarized in Supplementary Table S3 online. Using the linear predictor of the Cox regression model, we constructed a prognostic model, in which patients were classified into two risk groups (“high” and “low”) by determining whether log-risk = linear predictor > 0 or not, as previously reported^63^. The results of the prognostic model in two cohort studies were summarized in Supplementary Table S4 online. The log-rank test was used to evaluate whether two survival curves were statistically significantly different.

## Supplementary Information

Supplementary Table S1.

Supplementary Table S2.

Supplementary Table S3.

Supplementary Table S4.

## Data Availability

The datasets generated and/or analyzed during the current study are available from the corresponding author on reasonable request.
